# Supplementary material for: Noncured Graphene Thermal Interface Materials for High-Power Electronics: Minimizing the Thermal Contact Resistance
Source: Nanomaterials (Basel). 2021 Jun 28;11(7):1699. doi: 10.3390/nano11071699 (PMC8306163; doi:10.3390/nano11071699)
Supplement: Supplementary file 1 [file nanomaterials-11-01699-s001.zip › nanomaterials-1266677-supplementary.pdf]

nanomaterials MDPI

Supplementary Information

Noncured Graphene Thermal Interface Materials for High-Power Electronics: Minimizing the Thermal Contact Resistance

Sriharsha Sudhindra, Fariborz Kargar and Alexander A. Balandin *

Phonon Optimized Engineered Materials Center, Department of Electrical and Computer Engineering, University of California, Riverside, CA 92521 USA
* Correspondence: balandin@ece.ucr.edu; web-site: http://balandingroup.ucr.edu/

Thermal Conductivity and Thermal Contact Resistance Measurements

The produced thermal interface materials (TIMs) were tested using the industry standard ASTM D5470-06 TIM Tester (see the schematic in Figure S1). The equipment allowed determining the thermal conductivity and the thermal contact resistance of each compound at bond line thickness (BLT). The thermal contact resistance was obtained from the y-intercepts of the linear fitting regressions. The "bulk" thermal conductivity was extracted from the fitting plot using the formula:

$$Thermal\ Conductivity\ (Wm^{-1}K^{-1}) = \frac{1}{Slope} \times 100, \quad (1)$$

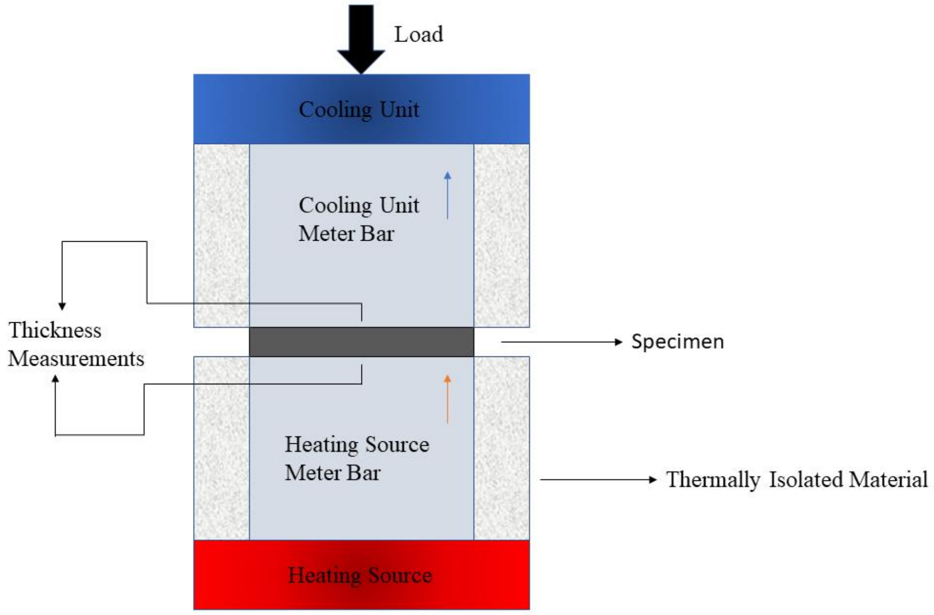

Figure S1. Schematic of LongWin ASTM D5470-06 TIM Tester used in this study to measure the thermal resistance of TIM applied between two metal plates. This standard follows the one-

dimensional heat conduction measurement technique. Heat flows from the heating plate near the heating source to the cooling plate near the cooling unit.

The described equipment was also used for determining the thermal contact resistance as a function of the copper plate surface roughness. Table S1 summarizes the obtained values of the "bulk" thermal conductivity and thermal contact resistance for each tested TIM.

Table S1. Thermal conductivity and thermal contact resistance of graphene TIMs.

Filler Loading (wt%)	Bulk Thermal Conductivity ($Wm^{-1}K^{-1}$)	Standard Error for Bulk Thermal Conductivity ($Wm^{-1}K^{-1}$)	Thermal Contact Resistance (Kcm^2W^{-1})	Standard Error for Thermal Contact Resistance (Kcm^2W^{-1})
0	0.176	0.004	0.681	0.090
2	0.237	0.100	0.183	0.330
5	0.330	0.030	0.149	0.160
10	1.578	0.110	0.051	0.020
15	1.748	0.180	0.011	0.040
18	2.483	0.200	0.035	0.020
20	2.645	0.180	0.075	0.020
30	3.326	1.200	0.147	0.100
40	4.221	1.100	0.234	0.060

Table S2 shows the obtained thermal contact resistance values for TIMs with 15 wt.% and 30 wt.% graphene filler loading and varying copper surface roughness.

Table S2. Thermal contact resistance of graphene TIMs with different plate roughness.

S_q Surface Roughness (μm)	Filler Loading (wt%)			
	15		30	
	$R_{C,tot}+R_{oil}$ (Kcm^2W^{-1})	Standard Error for $R_{C,tot}+R_{oil}$ (Kcm^2W^{-1})	$R_{C,tot}+R_{oil}$ (Kcm^2W^{-1})	Standard Error for $R_{C,tot}+R_{oil}$ (Kcm^2W^{-1})
0.05	0.082	0.269	0.430	0.510
1.2	0.379	0.040	0.850	0.010
2.5	0.734	0.096	1.001	0.080
3.1	0.875	0.234	1.070	0.210
